# One health, multiple challenges: The inter-species transmission of influenza A virus

**DOI:** 10.1016/j.onehlt.2015.03.001

**Published:** 2015-03-26

**Authors:** Kirsty R. Short, Mathilde Richard, Josanne H. Verhagen, Debby van Riel, Eefje J.A. Schrauwen, Judith M.A. van den Brand, Benjamin Mänz, Rogier Bodewes, Sander Herfst

**Affiliations:** a Department of Viroscience, Erasmus Medical Centre, The Netherlands; b School of Biomedical Sciences, University of Queensland, Brisbane, Australia

## Abstract

Influenza A viruses are amongst the most challenging viruses that threaten both human and animal health. Influenza A viruses are unique in many ways. Firstly, they are unique in the diversity of host species that they infect. This includes waterfowl (the original reservoir), terrestrial and aquatic poultry, swine, humans, horses, dog, cats, whales, seals and several other mammalian species. Secondly, they are unique in their capacity to evolve and adapt, following crossing the species barrier, in order to replicate and spread to other individuals within the new species. Finally, they are unique in the frequency of inter-species transmission events that occur. Indeed, the consequences of novel influenza virus strain in an immunologically naïve population can be devastating. The problems that influenza A viruses present for human and animal health are numerous. For example, influenza A viruses in humans represent a major economic and disease burden, whilst the poultry industry has suffered colossal damage due to repeated outbreaks of highly pathogenic avian influenza viruses. This review aims to provide a comprehensive overview of influenza A viruses by shedding light on interspecies virus transmission and summarising the current knowledge regarding how influenza viruses can adapt to a new host.

## Introduction

Influenza A viruses (Family Orthomyxoviridae) impose a large burden on both human and animal health worldwide. Influenza A viruses can be categorised into different subtypes based on genetic and antigenic differences in the two surface glycoproteins of the virus, the haemagglutinin (HA) and neuraminidase (NA). Wild waterfowl and shorebirds are the natural reservoirs of influenza A virus and can be infected with viruses harbouring combinations of 16 different HA subtypes and nine different NA subtypes. Recently, two novel influenza A virus subtypes (H17N10 and H18N11) have been identified in rectal swabs collected from the little yellow-shouldered bat [*Sturnira lilium*] and the flat-faced fruit-eating bat [*Artibeus jamaicensis planirostris*] [1], [2], [3]. Influenza viruses of this subtype have not been isolated from any other animal order and it is unknown whether these viruses might be able to cross the species barrier. In contrast, there is significant inter-species transmission of influenza viruses from waterbirds, such that animals ranging from domestic poultry to humans can also become infected. Accordingly, infection with influenza virus has wide-reaching ramifications. For example, whilst some influenza virus strains are largely asymptomatic in chickens (and are hence referred to as low pathogenic avian influenza [LPAI] viruses) others cause severe disease in chickens that is often fatal within 48 h (and are hence referred to as highly pathogenic avian influenza [HPAI] viruses). Outbreaks of HPAI viruses can cause devastation for the poultry industry resulting in the mass slaughter of millions of birds [4]. Similarly, outbreaks of influenza viruses amongst thoroughbred horses have disrupted numerous race meetings and resulted in the death of infected horses [5]. In humans, seasonal influenza viruses are a significant cause of morbidity and mortality and constitute an economic burden of $10.4 billion dollars per year in the U.S.A. alone [6]. The diversity and complexity of influenza virus infections across so many different animal species suggests that a one-health approach is the only comprehensive way to reduce the burden of disease. Here, we seek to highlight how influenza viruses spread from their natural avian hosts to mammals, and what the virus needs to overcome in order to ensure the success of these inter-species transmission events. We highlight the consequences that this inter-species transmission has, not only for human health, but also for the health of wild animals and the success of industries such as poultry farming.

## Influenza A viruses in animals

### Wild birds as a reservoir of LPAI viruses

**Fig. 1:**
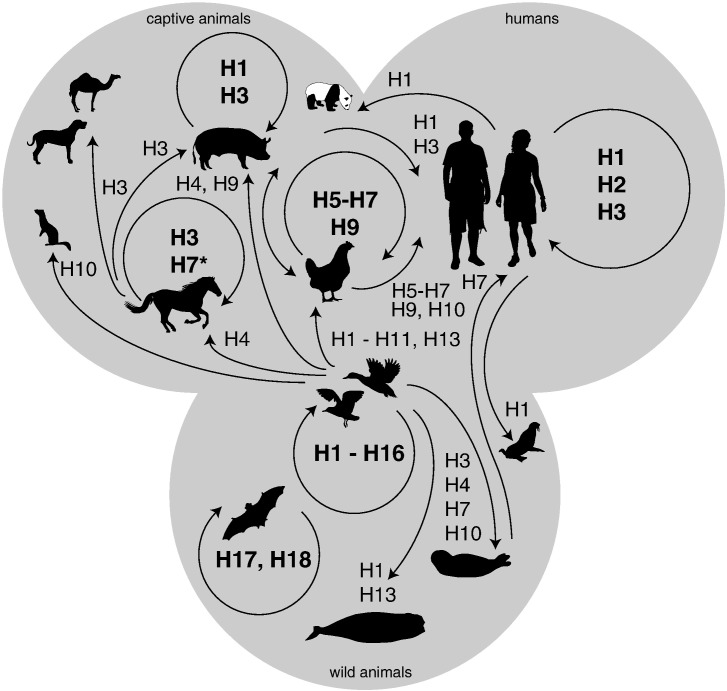
Reservoirs and inter-species transmission events of low pathogenic avian influenza viruses. Wild birds, domestic birds, pigs, horses, humans and bats maintain their own influenza A viruses (arrow in circle, subtype in bold). Spill-over events occur occasionally, most frequently from wild birds (arrow straight, subtype normal font). *H7N7 virus emerged amongst horses in the 1950s but is currently thought to be extinct.

Waterbirds and shorebirds of the orders *Anseriformes* (mainly ducks, geese and swans) and *Charadriiformes* (mainly gulls, terns and waders) are considered the natural host reservoirs of LPAI viruses [7] (see Fig. 1). In wild birds LPAI viruses predominantly infect epithelial cells of the intestinal tract [8], [9] and are subsequently excreted in the faeces. However, infection of wild birds with LPAI viruses is typically sub-clinical and occurs in the absence of obvious lesions [10], [11], [12]. Every year, LPAI viruses cause outbreaks amongst waterbirds. These outbreaks are most commonly associated with the increased presence of juvenile, immunologically naïve birds in the population and occur during migration when contact rates between, and within, populations are high [13]. The relatively high virus prevalence in waterbirds may be due, in part, to virus transmission through the faecal–oral route via surface waters [7].

### Transmission of LPAI viruses to marine mammals

Influenza viruses from waterbirds can cross the species barrier and infect numerous other species (see Fig. 1). A recent example of bird-to-animal transmission is the mortality amongst harbour seals [*Phoca vitulina*] of the North-European coastal waters following infection with the LPAI H10N7 virus [14], [15], [16]. Various outbreaks of LPAI H3, H4 and H7 viruses causing severe respiratory disease and mortality amongst harbour seals have also occurred in the past decades along the New England coast of the United States of America [17], [18], [19], [20]. The exact transmission route between seals is unknown but it is likely to occur via the respiratory route, most probably whilst the seals are resting on land. It is currently unknown if adaptation of influenza viruses from waterbirds is needed to allow the virus to infect and transmit amongst seals. In addition to seals, LPAI viruses have been isolated from a long-finned pilot whale [*Globicephala melas*] and Balaenopterid whales (species unknown) [21], [22], and serological evidence for infection was reported in various other marine mammal species (for review see [18]). However, the available data is very limited and it is remains unclear if LPAI viruses can also cause outbreaks of disease in other marine mammals similar to harbour seals.

### Transmission of LPAI viruses to domesticated animals

In addition to transmission to marine mammals, LPAI viruses can also cross the species barrier and infect domesticated mammals and birds (see Fig. 1). Swine [*Sus scrofa domesticus*] have become infected with H4 and H9 viruses sporadically, whilst LPAI H1 and H3 viruses are endemic in pigs. Disease manifestations can range from as acute respiratory tract disease to an inapparent infection [23]. Traditionally, swine infections were considered to be of particular importance because swine respiratory epithelial cells were thought to express both α2,3- and α2,6-linked sialic acids [23]. In general, human influenza viruses use α2,6-linked sialic acids as a receptor, whereas avian influenza viruses attach predominantly to α2,3-linked sialic acids [24]. Accordingly, the presence of both α2,6- and α2,3-linked sialic acids would mean that swine may potentially be infected by both avian and human viruses [23]. Due to its segmented genome, when two different influenza viruses infect a single host, virus gene segments can be inter-changed such that a novel so-called ‘reassortant’ virus is produced. Pigs were thus traditionally thought to act as a ‘mixing vessel’ and facilitate the generation of novel reassortant influenza viruses. However, recent studies suggest that there is in fact limited α2,3-linked sialic acid on swine tracheal cells [25]. Rather, perhaps of greater importance for one health, is the presence of large numbers of pigs in close proximity to other animal species [26], increasing the risk of an inter-species transmission event.

Influenza viruses currently circulating in mammalian species, including dogs [*Canis lupus familiaris*] and horses [*Equus ferus caballus*], are also thought to have derived from avian influenza viruses [27], [28], [29]. Viruses of the H3N8 and H3N2 subtypes are currently circulating amongst dogs [30]. Whilst the H3N2 virus is a recent direct spill-over from birds, the H3N8 virus has been circulating amongst horses since at least the 1960s and was established in the dog population in the end of the last century or the early 2000s [27], [31], [32], [33], [34]. Both viruses can cause severe respiratory disease in dogs [34], [35]. In horses, H3N8 viruses were first detected in the 1960s and have subsequently become endemic [33], [36]. The H7N7 virus emerged amongst horses in the 1950s but is currently thought to be extinct [36]. Interestingly, the H3N8 virus was also detected in a Bactrian camel [*Camelus bactrianus*] [37].

Terrestrial poultry can also become infected with numerous different LPAI viruses. A subset of the LPAI virus strains (namely H5, H6, H7, H9 viruses) that infect poultry can subsequently become endemic in this population [38], [39]. Indeed, LPAI viruses appear to adapt upon infection of terrestrial poultry, as LPAI viruses isolated from wild birds do not replicate efficiently if used to experimentally infect poultry [40]. In poultry LPAI viruses typically cause limited clinical signs [41]. However, unlike in water birds, LPAI viruses predominately infect respiratory epithelial cells [42]. Accordingly, it is thought that LPAI virus strains can also be transmitted amongst terrestrial poultry, roaming in dry pens at high densities, via respiratory droplets and aerosols [43]. However, perhaps the most significant feature of LPAI virus infection of poultry is that in these species that some LPAI viruses are able to evolve into HPAI viruses.

### HPAI viruses in domestic birds

In terrestrial poultry, LPAI viruses of the H5 and H7 subtypes can subsequently evolve to become HPAI viruses by the insertion of a multi-basic cleavage site in the viral HA. The HA of LPAI viruses is cleaved by trypsin-like proteins, expressed in the intestinal and respiratory tracts of birds. However, the presence of the multi-basic cleavage site in the HA of HPAI viruses enables these viruses be cleaved by the furin family of enzymes. These enzymes are distributed throughout the body hence enabling HPAI viruses to cause a systemic viral infection [43]. To date, this evolution to a highly pathogenic phenotype has only been observed in H5 and H7 influenza viruses. It remains unclear whether this represents a unique feature of these viral strains, or if this is simply a chance event. HPAI viruses typically cause a fatal and systemic infection in terrestrial poultry characterized by severe necrosis and inflammation of multiple organs, often with virus replication in endothelial cells [44]. Upon its evolution in terrestrial poultry, HPAI viruses can once again cross the species barrier and be transmitted to other avian and mammalian species.

### HPAI viruses in wild birds

The HPAI H5N1 virus is unusual in so far as it has infected and caused mortality in a variety of different waterbirds [45], [46]. For example, in 2005 there was an outbreak of the HPAI H5N1 virus in China that resulted in a 10% decrease in the global population of bar-headed geese [47]. Wild birds most probably become infected with HPAI H5N1 virus via the inhalation of infectious droplets or aerosols and/or contact with contaminated water [43]. There is also evidence to suggest that wild birds of prey become infected with HPAI H5N1 viruses following consumption of infected carcasses [48]. In ducks, the extent of disease observed following infection with HPAI H5N1 viruses varies depending on the duck species in question [49]. For example, whilst tufted ducks (*Aythya fuligula*) and pochards (*Aythya farina*) display neurological signs upon infection with HPAI virus A/turkey/Turkey/1/2005(H5N1), no clinical signs were observed in mallard (*Anas platyrhynchos*), common teals (*Anas crecca*), Eurasian wigeon (*Anas penelope*) or gadwalls (*Anas strepera*) infected with same dose and strain of virus [49]. Indeed, in light of these differences the mallard duck is considered the most likely candidate for a long-distance vector of HPAI H5N1 viruses, as this species excretes abundant amounts of virus without showing clinical signs or being debilitated by the disease [49]. Similarly, the high seroprevalence of H5N8-specific antibodies in various duck species suggest that these animals are able to survive the initial virus infection and may be involved in the epidemiology of this lineage of HPAI H5 viruses [50].

### HPAI viruses in non-human mammals

The HPAI H5N1 virus has also been detected in various animals living in captivity, including tigers [*Panthera tigris*], leopards [*Panthera pardus*], a dog, cats [*Felis catus*], Owston's palm civets [*Chrotogale owstoni*] and swine (for review see [51]). H5N1 viruses have also been detected in a free-living stone marten (*Mustela foina*) and an American mink (*Mustela vison*) [52], [53] (for review see [51]). Infection of carnivores occurs most likely via the ingestion of infected poultry or wild birds, although there has been evidence of horizontal transmission of HPAI H5N1 virus amongst tigers [54]. Interestingly, infection of carnivores with the H5N1 virus caused severe respiratory and systemic disease, often with involvement of the central nervous system, whilst no severe disease was observed in swine, both after natural or experimental infection (for review see [51]).

**Influenza A viruses in reptiles**

Antibodies were detected against multiple influenza virus strains in captive species of reptiles and amphibians, whilst influenza virus RNA was detected in captive crocodilians [55], [56], [57]. In addition, in vitro and in ovo experiments suggest that alligators are susceptible to infection with avian influenza viruses [58]. Since birds are evolutionarily closely related to reptiles, identification of divergent viruses that circulate amongst species of this order might provide interesting information about the evolutionary history of influenza viruses.

## Human influenza A virus infections

To date, the four best described influenza A virus pandemics amongst humans are the 1918 H1N1 ‘Spanish flu’, the 1957 H2N2 ‘Asian flu’, the 1968 H3N2 ‘Hong Kong flu’ and the 2009 H1N1 pandemic. These pandemics have been of varying severity and origin. For example, whilst the 1918 pandemic virus is thought to be avian descent, the 1968 H3N2 virus was derived from a reassortment event between the circulating human H2N2 virus and the HA and PB1 genes from avian influenza virus strain [59]. Although some of these pandemic viruses have become extinct (such as the 1957 H2N2 virus), others have become established in the human population and are responsible for seasonal outbreaks of influenza virus observed every year [59]. The two seasonal influenza A virus strains currently circulating in humans (pH1N1 and H3N2) cause approximately 500,000 deaths worldwide, with infection typically manifesting as an acute respiratory tract infection [60]. Thus, influenza viruses, and in particular influenza virus strains that have been recently introduced into the human population, represents a continuous threat to human health. The vast majority of zoonotic events are restricted to sporadic individual cases, without evidence of sustained human-to-human transmission. However, the acquisition of human-to-human transmissibility by a zoonotic virus could mark the beginning of a new influenza virus pandemic.

## Avian influenza viruses that directly infect humans

### Zoonotic events

**Fig. 2:**
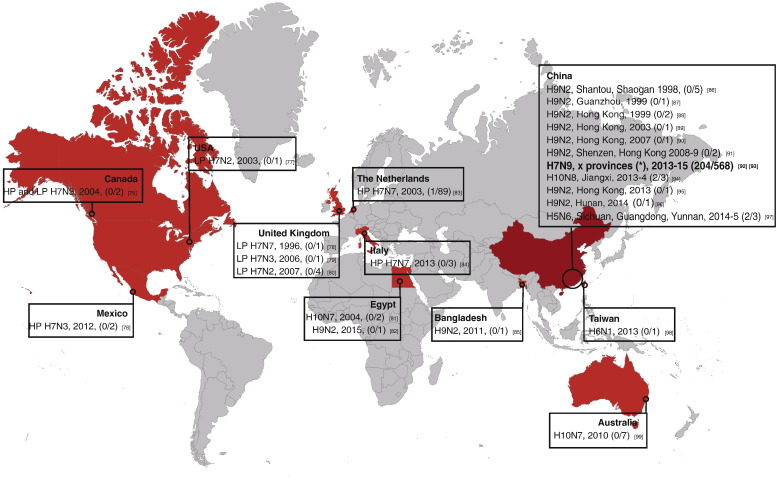
Zoonotic events caused by influenza viruses originating from avian species. The countries where avian zoonotic infections were recorded are indicated in shades of red. * provinces of China: Shanghai, Beijing, Hong Kong, Anhui, Fujian, Jiangsu, Jiangxi, Guangdong, Guizhou, Henan, Hunan, Hebei, Shandong, Zheijang. Additionally, two cases were imported to Taiwan from mainland China. The intensity of the colour correlates with the intensity of the reported cases. In brackets are indicated the number of fatalities against the number of confirmed cases.

**Fig. 3:**
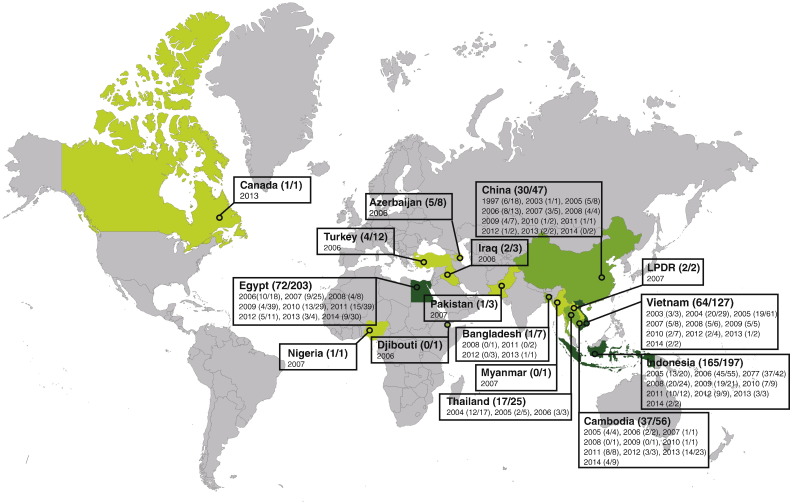
Zoonotic events caused by influenza H5N1 viruses. The countries where human cases of H5N1 virus infection were recorded since 1997 are indicated in shades of green. LPDR: Lao People's Democratic Republic. The intensity of the colour correlates with the intensity of the reported cases. In brackets are indicated the number of fatalities against the number of confirmed cases.

Adapted from [70].

Since the first report of transmission of HPAI H5N1 viruses to humans in 1997, it became clear that as well as infecting a variety of other species, avian influenza viruses could also infect humans [61], [75], [76], [77], [78], [79], [80], [81], [82], [83], [84], [85], [86], [87], [88], [89], [90], [91], [92], [93], [94], [95], [96], [97], [98], [99]. To date, avian influenza viruses of subtypes H5, H6, H7, H9 and H10, have crossed the species barrier and caused human infections (see Fig. 2, Fig. 3). Of particular concern are the HPAI H5N1 and LPAI H7N9 virus strains due to the frequency of zoonoses and/or the severity of disease they cause (see Fig. 2, Fig. 3).

HPAI H5N1 viruses were first detected in humans in Hong Kong in 1997. During this outbreak 18 people were infected with six fatalities [62]. In 2003 and 2004, H5N1 viruses spread from East Asia to Southeast Asia, where sporadic human infections were recorded along with outbreaks in terrestrial and aquatic poultry [63]. Following an outbreak of HPAI H5N1 influenza virus amongst waterfowl at Qinghai lake, China, in 2005 [64], H5N1 viruses spread from China [65], westward to India [65], to Europe [66], and Northern and Central Africa [67]. In these countries, HPAI H5N1 viruses were detected in poultry, many species of wild birds and mammals. HPAI H5N1 viruses currently represent a major threat to both human and animal health owing to their unique characteristics. For example, H5N1 viruses represent the only lineage of HPAI viruses that are enzootic in poultry in many parts of the world, and this has been the case for over a decade. Quarantining of infected farms, and culling of infected or potentially exposed poultry causes severe damage to the poultry industry and animal health worldwide, with an estimation of 400 million of birds culled and economic losses equivalent to US$20 billion [68]. This continued circulation in poultry led to a diversification in different genetic clades, either by reassortment or by accumulation of point mutations (for a review, see [69]). HPAI H5N1 viruses are also exceptional in the diversity of species that they infect, by the high incidence of zoonotic events and the severity of human infections that they cause. Indeed, since 1997 HPAI H5N1 viruses have been reported to infect almost 700 people, with a case fatality rate close to 60% [70] (Fig. 3). Although a few clusters of human cases were reported, no sustained human-to-human transmission has been recorded [71]. Rather, human infections typically occur following close contact with infected birds [43]. Live poultry markets and backyard production with poly-culture farming (i.e. farming more than one animal species in the same location) have thus played an important role in the inter-species transmission of HPAI H5N1 viruses and may help explain, in part, the preponderance of HPAI H5N1 virus infections in Asia and North Africa [72], [73], [74].

In addition to HPAI H5N1 viruses, the emergence of a novel LPAI H7N9 virus in humans in March 2013 has been a cause for concern. H7N9 viruses were first detected in China and the number of human cases peaked in April 2013 [93], [100]. The incidence of infection subsequently declined, most probably due to the implementation of control strategies such as the closure of live bird markets and increased public awareness. Indeed, it is thought that the closure of live poultry markets in Shanghai reduced the mean daily number of human H7N9 virus infections by 99% [101]. A rise in the number of cases occurred in the Northern Hemisphere winter of 2013/2014, constituting a second wave, and cases are still being detected in China, resulting in the third wave [93]. Both the second and third waves coincided with the influenza season in China. Since the first wave of zoonotic events in 2013, more than 500 laboratory-confirmed human infections, with a case fatality rate of 36%, have been reported [93]. As a LPAI virus, H7N9 viruses do not cause severe disease in poultry and therefore circulate undetected, making it more difficult to contain and/or eradicate outbreaks. Cluster of human infections with H7N9 have been reported [102], demonstrating that human-to-human transmission may have occurred. However, H7N9 viruses do not so far have the ability to transmit efficiently between humans.

### Pathogenesis and transmission

The severity of the disease caused by avian zoonotic influenza viruses is diverse. Symptoms range from conjunctivitis (often associated with the H7 subtype) to pneumonia, acute respiratory distress syndrome (ARDS) and encephalitis [107], [108], [109], [110], [111], [112], [113], [114], [115], [116], [117], [118], [119], [120], [121], [122], [123], [124], [125], [126], [127], [128], [129], [130], [131], [132], [133], [134], [135], [136], [137], [138], [139], [140], [141], [142], [143], [144], [145], [146], [147], [148], [149], [150], [151], [152], [153], [154], [155], [156], [157], [158], [159], [160], [161], [162], [163], [164], [165], [166], [167], [168], [169], [170], [171], [172], [173], [174], [175], [176], [177], [178], [179], [180], [181], [182], [183], [184], [185], [186], [187], [188], [189], [190], [191], [192], [193], [194], [195], [196], [197], [198], [199], [200], [201], [202], [203], [204], [205], [206], [207], [208], [209], [210], [211], [212], [213], [214], [215], [216], [217], [218], [219], [220]. H7N9 and HPAI H5N1 stand out by the severity of the disease they cause. Indeed, human infections with H7N9 and HPAI H5N1 are typified by severe respiratory insufficiency and the development of ARDS [103]. Histologically this is characterized by diffuse alveolar damage (DAD) with necrosis of alveolar epithelium, intraluminal oedema, fibrin, haemorrhage, neutrophils and increased numbers of alveolar macrophages [104]. In the later stage there is also type II hyperplasia, interstitial infiltration of lymphocytes and plasma cells and fibrosis [105]. The mechanisms underlying influenza virus-induced DAD and the associated respiratory insufficiency are both complex and multi-faceted (for a more extensive list of the key players in damage see [106], [107]). One striking feature of these severe and sometimes fatal influenza virus infections is the high levels of pro-inflammatory cytokines produced by alveolar epithelial cells and leukocytes [108]. This has led to the theory that a ‘cytokine storm’ (i.e. an uncontrolled pro-inflammatory response) contributes to disease severity [108]. Interestingly, in vitro infection of primary human macrophages and alveolar epithelial cells suggests that whilst H7N9 viruses are more potent inducers of pro-inflammatory cytokines than H1N1 viruses, neither virus strain is as potent as HPAI H5N1 viruses [109]. This may offer a potential explanation for the differential mortality observed between H7N9 and HPAI H5N1 infections [109]. However, it is important to note that exactly how these pro-inflammatory cytokines damage alveolar function remains unclear [106]. Damage may occur indirectly via the recruitment of specific leukocyte subsets to lung. Alternatively, specific cytokines may induce epithelial cell death and hence facilitate pulmonary oedema. The relative important of these different mechanisms during infection remains an important area for future research.

The severity of HPAI H5N1 virus infections in humans compared to other influenza virus strains may be due, in part, to the receptor tropism of these viruses. As described above, the receptor tropism of influenza viruses is determined by the HA, which can bind to either α2,6-linked sialic acid (typically seen with human-derived HAs) or α2,3-linked sialic acid (typically seen with avian-derived HAs), or both. In humans, α2,6-linked sialic acids are strongly expressed in ciliated epithelial cells (such as those present in the upper respiratory tract, trachea and bronchus) whilst within the alveolus type II pneumocytes express predominantly α-2,3 linked sialic acids [110], [111]. Accordingly, virus histochemistry has shown that HPAI H5N1 viruses attach predominantly to non-ciliated cuboidal cells (club cells) in the bronchiole and type II pneumocytes and alveolar macrophages in the alveoli [24], [112], [113], [114], [115]. It is therefore tempting to speculate that avian viruses can cause severe disease in humans by infecting the cells in the lower respiratory tract, but that this lower respiratory tract binding pattern limits their transmissibility. However, this association between the attachment pattern, disease pathogenesis and transmission efficiency in humans is not always so straightforward. For example, unlike HPAI H5N1 viruses, H7N9 viruses attach to epithelial cells of both the human upper and lower respiratory tract [116]. Although the attachment to cells of the lower respiratory tract corresponds to the severe disease caused by H7N9 viruses in humans, the attachment to ciliated epithelial cells of the upper respiratory tract has not resulted in efficient aerosol transmission amongst humans nor between ferrets, an animal model for influenza virus transmission [117], [118], [119]. In addition, HPAI H5N1 influenza viruses with mutations that result in attachment to ciliated epithelial cells of the upper respiratory tract, did not result in aerosol transmission in a ferret model [120], [121].

**Fig. 4:**
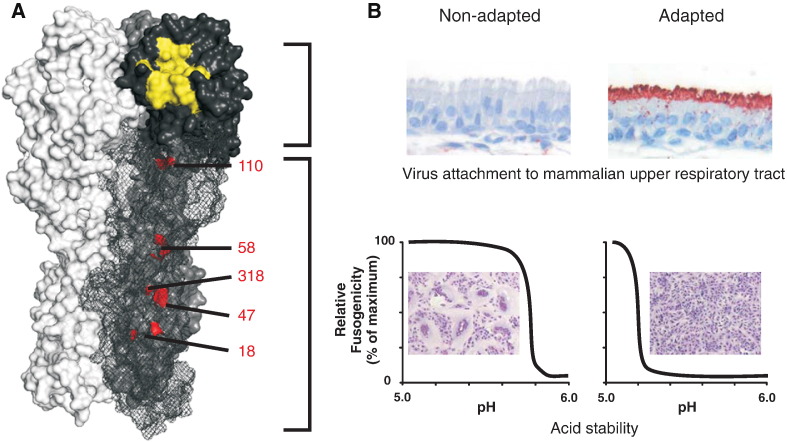
Adaptation of the hemagglutinin protein in mammals. A) Cartoon representation of a model of the trimer structure HA of A/Indonesia/5/2005 (PDB ID: 1JSM). The momomers have different colours for clarity. Highlighted in yellow is the receptor binding site, that facilitates attachment to the sialic acid receptors on the host cell. Mutations in, or in close proximity of the receptor binding site, together with the presence or absence of potential N-linked glycosylation sites, affect the preference and avidity of binding to α2,3-linked or α2,6-linked sialic acid receptors. Amino acids (position 18, 110, 318, 47 (HA2) and 58 (HA2) (H3 numbering), which have been shown to affect HA-stability, and associated transmission in mammals are highlighted in red. B) Influenza virus phenotypes which are associated with adaptation to mammals, shown for non-adapted and adapted avian influenza viruses. Top panel: Attachment of inactivated influenza viruses to ciliated epithelial cells in ferret upper respiratory tract. Bottom panel: acid stability of HA proteins as measured in syncytia formation assays after exposure to indicated pH.

The above studies clearly indicate that attachment to ciliated epithelial cells of the upper respiratory tract is likely to be necessary, but not sufficient, for efficient viral transmission amongst humans. To further define what limits the transmissibility of avian viruses amongst humans, studies assessing the airborne transmissibility of experimentally generated HPAI H5N1 viruses amongst ferrets have been performed [121], [122]. In addition to mutations that were intentionally introduced in the receptor binding site (RBS) of HA to enhance binding to α2,6-linked sialic acid and decrease binding to α2,3-linked sialic acid, both studies found two other mutations in the viral HA during ferret passage (see Fig. 4). These mutations affected the stability of HA (see below), or resulted in the loss of a potential N-linked glycosylation site close to the receptor binding site, thereby increasing virus binding to sialic acid receptors [122], [123].

After binding of the HA to sialic acid receptors on the host cells, virus particles enter the cell through a process called receptor-mediated endocytosis. The acidic environment in the endosome triggers a conformational change of the HA, enabling fusion of the viral and endosomal membrane, and the subsequent release of the viral genome in the cytoplasm. The pH at which the HA undergoes this conformational change varies between the HA subtypes and virus strains [124]. Interestingly, the pH of fusion of many avian influenza viruses is higher (less acidic) than that of human influenza viruses [125]. The two mutations that were identified in the HA of airborne H5N1 viruses (H110Y, T318I, H3 numbering) stabilized the HA protein, as demonstrated by a lower pH of fusion, and a higher temperature stability [122], [123].

Dozens of other mutations that modulate the pH of fusion have been described, some of which have been proven to change the replication and transmission in mammals (for an extensive review see Mair et al., 2014) [126]. For example, lowering the pH threshold of fusion by an H18Q (HA1) or K58I (HA2) substitution in an avian H5N1 HA resulted in enhanced replication in the upper respiratory tract of ferrets [127], [128]. The requirement for a low pH of fusion may also explain the lack of sustained transmission of H7N9 between humans, since the pH of fusion of H7N9 HA is relatively high compared to the HA of human influenza viruses [129].

Although more in vitro data is accumulating on HA stability, one must remember that these properties may merely be a surrogate for another (as yet unknown) phenotype. This phenotype may be the stability of the HA in aerosols, resistance to drought, stability in mucus or altered pH in the host environment. For example, studies in humans have shown that the respiratory tract environment is mildly acidic, with a mean pH of 6.3, but with a range between pH 5 and 8 [130]. Therefore, it is possible that an unmodified avian influenza virus with a relative unstable HA (i.e. pH of fusion around 5.6, as has been demonstrated for H5N1), might be inactivated by a premature, pH-triggered conformational change before it can bind and enter its target cell in the respiratory tract.

**A balanced approach**

After virus replication, the receptor-destroying activity of the NA protein facilitates the release of newly formed virus particles from the cell surface. An optimal interplay between the receptor-binding of the HA and receptor-destroying activity of the NA is required [131]. Changes in the receptor binding activity can disturb this functional balance, which can be restored by compensatory mutations in the HA, NA or both. One striking example of this balance is the concomitant glycosylation of the HA and NA stalk truncature as the result of adapting to terrestrial birds [132]. It has also been hypothesised that a fine balance between HA and NA functions is necessary for progeny virions to be released as single particles, which might be beneficial for airborne transmission [131]. Providing definitive experimental evidence to support this hypothesis remains an important area for future studies.

After release of the viral genome in the cytoplasm, and transportation of the gene segments to the nucleus, the attached viral polymerase complex consisting of the basic polymerase 2 (PB2), basic polymerase 1 (PB1) and acidic polymerase (PA), needs to produce a high amount of viral genomes and proteins for the generation of new virus particles. The high copy rate of the viral genome also ensures the fast accumulation of (potentially beneficial) mutations, a hallmark of a host species switch by RNA viruses. However, the polymerase activity of avian influenza viruses is strongly restricted in mammalian cells [133]. Avian influenza viruses normally replicate at a temperature of ~ 41 °C, i.e. the temperature in the avian intestinal tract, and after transmission to humans, they need to adapt to replicate at ~ 33 °C, the accepted temperature for efficient replication in the mammalian upper respiratory tract.

PB2 was the first polymerase protein in which a mutation that helped overcome this host restriction was identified [134]. Whilst avian influenza A virus isolates contain almost exclusively a glutamic acid at position 627 of the polymerase PB2 protein (PB2-E627), this position is frequently mutated to a lysine in human-derived isolates. Mutation to PB2-E627K is accompanied by improved viral replication at low temperatures [135], [136] and enhanced binding of the viral polymerase to the nucleoprotein in human cells [137], thereby also evading recognition by the pathogen sensor RIG-I [138]. In addition, PB2-E627K was shown to be required to confer airborne transmissibility to a HPAI H5N1 virus between ferrets [121], [123]. Notably, fatal infections of humans with avian viruses of the H5N1 and H7N9 subtype that retained the avian like glutamic acid at position 627 of PB2 are frequent [139]. These data suggest that PB2-E627K is not always strictly necessary for avian viruses to cross the species barrier and infect mammals. Recently, a PB2-K526R substitution was found to increase polymerase activity, especially in concert with PB2-E627K [140]. Interestingly, this mutation was found in all subtypes of seasonal influenza viruses, as well as in human isolates of H5 and H7 infections, but is rare in avian isolates. In guinea pigs PB2-D701N could substitute for the lack of PB2-E627K for contact as well as aerosol transmission [136], possibly by improved nuclear import of the polymerase subunit PB2 [141]. Other mutations known to play a role in host adaptation (increased polymerase complex activity, or virulence) have been described elsewhere [142], [143], [144], [145], [146], [147], [148], [149], [150], [151], [152], [153], [154], [155], [156], [157], [158].

In addition to PB2, features of avian PB1 may also restrict the human-to-human spread of avian influenza viruses. For example, for the ferret airborne-transmissible HPAI H5N1 virus, it was shown that, together with PB2-E627K, PB1-H99Y increased polymerase activity, by improving the balance between transcription and replication, a requirement for efficient airborne transmission between ferrets [123]. Other mutations in the polymerase complex protein, nucleoprotein (which binds to the viral genome to prevent degradation by host cell enzymes) and the non-structural protein NS2/NEP may also help avian influenza viruses overcome replication restrictions mammalian hosts. Indeed, escape mutants in the viral nucleoprotein can reduce sensitivity of avian influenza viruses to the human protein MxA which possess strong antiviral activity and helps limit viral replication [159], [160], [161], [162]. Whilst the effects of most of these mutations on airborne transmissibility have not been studied, it is anticipated that these mutations, alone, or in concert with other (compensatory) mutations may affect transmissibility by improving viral polymerase activity in mammals.

**Fig. 5:**
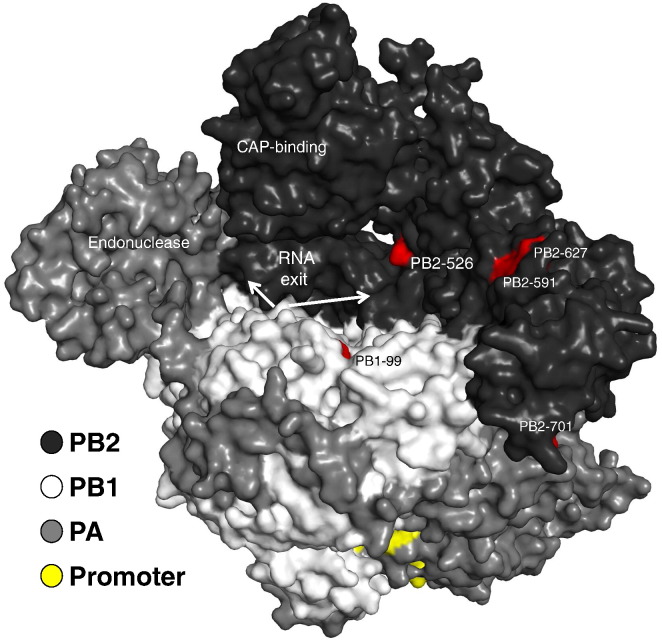
Adaptation of the polymerase complex. The programme PyMOL was used to assign the indicated subunits PB2, PB1, PA and the influenza A virus promoter in dark grey, grey, light grey and yellow, respectively. Characterized domains, as well as amino acids relevant for airborne transmission between mammals are indicated. The promoter is bound by parts of all three polymerase subunits. The template, as well as the nascent strand exit the polymerase from a cavity, presumably in two different directions. The structural model of A/little yellow-shouldered bat/Guatemala/060/2010 (H17N10) served as a basis (PDB code: 4WSB).

The recently solved structure of the influenza polymerase bound to its promoter [163] may help shed light on location and interplay of known adaptive mutations as well as those yet to be identified. A proposed RNA exit channel located between the PB1-subunit and the cap-binding domain contains most of the adaptive mutations of PB2. For human influenza viruses, only PB2-E627K, PB2-D701N, PB1-H99Y and likely PB2-Q591R could be conclusively associated with efficient transmission and therefore successful adaptation (see Fig. 5) [121], [136], [158], [164]. As these amino acid positions are located in close proximity with each other, it remains to be shown if amino acid changes in other parts of the polymerase can lead to a similar level of adaptation to mammals.

## Influenza viruses that infect humans via an intermediate mammalian host

**Fig. 6:**
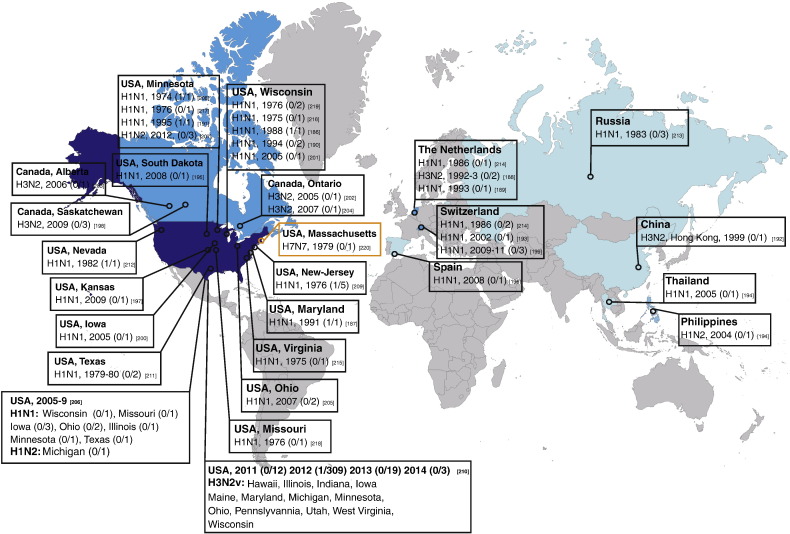
Zoonotic events caused by influenza viruses originating from mammalian species. The countries where swine zoonotic infections were recorded are indicated in shade of blue. The orange box indicates the only reported human infection with a seal-origin influenza virus. The intensity of the colour correlates with the intensity of the reported cases. In brackets are indicated the number of fatalities against the number of confirmed cases.

In addition to being directly infected by avian influenza virus strains, humans can also be infected with influenza virus strains that have passed through an intermediate mammalian host (e.g. swine) (see Fig. 6). Indeed, prior to the outbreak of HPAI H5N1 viruses in 1997, the dogma was that infection via an intermediate host was the *only* path by which a new influenza virus strain could enter the human population. Several influenza virus lineages have become enzootic in swine populations upon transmission of influenza viruses from birds and humans to pigs [165]. Influenza viruses of H1N1, H1N2 and H3N2 subtypes, as well as reassortants between these enzootic influenza viruses, have then crossed the species barrier and caused human infections [186]. Perhaps the most striking example of a virus that, via swine, can infect humans was the 2009 pandemic H1N1 virus. This virus had a unique gene constellation combining the genes from human, avian and swine influenza viruses [166]. The 2009 pandemic H1N1 virus was first detected in humans in February, 2009 [167]. This virus then rapidly spread across the globe, infecting between 10 and 20% of the world's population and eventually replacing the human seasonal H1N1 virus [168]. Consistent with this efficient transmission, the 2009 pandemic H1N1 virus was able to bind to α2,6-linked sialic acid and to a lesser extent α2,3-linked sialic acid [169]. However, experimental studies suggest that transmission determinants of this virus resided in the matrix gene segment (which encodes the protein (M) matrix (M1) and proton channel (M2) proteins) as well as the NA [170], [171], [172]. It was suggested that M1 and/or M2 affect the functionality of NA by changing the virion morphology [173]. Surprisingly, the pandemic influenza 2009 H1N1 virus did not contain the mammalian adaptation residues 627K and/or 701N. When the substitutions E627K or D701N were introduced in pandemic influenza 2009 H1N1, no differences in virulence or transmissibility were observed [174]. The lack of these mammalian adaptation markers in PB2 were partially compensated by substitutions G590S/Q591R, which may affect interaction with viral and/or cellular factors and promote virus replication in mammals [158].

Despite a high rate of transmission, the mortality rate of the 2009 pandemic H1N1 virus was relatively low: approximately 29 deaths per 100,000 infections [175]. Interestingly, there were marked differences in the severity of disease observed during the pandemic [176]. For example, whilst most patients displayed mild, self-limiting symptoms some developed ARDS reminiscent of HPAI H5N1 virus infection [177]. Indeed, some patients even displayed signs of CNS disease following infection [178], [179]. The development of severe respiratory disease was associated, in part, with the presence of specific viral mutations. For example, the D222G mutation in the HA of the pH1N1 virus was associated with some severe and fatal infections [180]. This is thought to relate to an increased preference of these viruses to bind to α2,3-linked sialic acid and thus infect of cells within the lower respiratory tract [181]. Severe disease was also associated with the presence of co-morbidities such as asthma, obesity and pregnancy [182], [183]. Indeed, many of the identified risk factors were related to a compromised immune system or aerodynamic ventilation problems. Interestingly, most patients with pH1N1 infection were (young) adults with a median age of 36 years [184]. This is in direct contrast to seasonal influenza virus infections, where it is those in the extreme of age who are at the highest risk of severe disease. Monsalvo and colleagues [185] offer an elegant explanation as to why, at least some, young adults developed severe disease. Monsalvo et al. [185] demonstrated that during the 2009 pandemic many middle aged adults possessed cross-reactive, but not protective, antibodies to the virus. Severe disease was associated with the ability of these antibodies to form immune complexes and activate the complement cascade within the respiratory tract [185]. Thus, whilst the presence of cross-reactive antibodies can protect against disease development, non-protective cross-reactive antibodies represent an important host factor that exacerbates disease development.

In addition to the 2009 pandemic H1N1 virus, numerous other influenza virus strains have infected humans via an intermediate swine host. The vast majority of these reported human cases are located in the United States (see Fig. 6). Of particular interest is the H3N2 variant (H3N2v) variant virus. This virus is a reassortant virus which contains the M gene of the 2009 pandemic H1N1 virus and the remaining genes of the North American swine influenza virus lineage [221]. Since the first documented cases of human H3N2v virus infections in 2011, over 350 human infections with swine H3N2v virus have been reported, including one death of a person with multiple underlying conditions [222]. Typically, these infections are associated with close contact with pigs during activities such as care giving, visiting live-animal markets or fairs [223]. However, a limited number of cases reported no contact with swine, indicating the possibility of limited human-to-human transmission of H3N2v virus [224]. Indeed, experimental studies in ferrets show that H3N2v is capable of replicating in the upper respiratory tract and transmitting efficiently to naïve ferrets via respiratory droplets [225].

## Sustained transmission in humans

Finally, it is important to recognise that when a new influenza virus strain enters the human population the virus will continue to adapt and change. For example, the 2009 pandemic H1N1 virus, despite already being transmissible, evolved towards a more acid stable virus due to an E47K (HA2) substitution which gave rise to a higher infectivity in ferrets [226]. It remains to be determined how other influenza virus strains have evolved during the course of a pandemic. Reassortment with contemporary influenza viruses is also possible in order to improve viral replication and/or transmissibility. It was demonstrated that reassortment between 2009 pandemic H1N1 virus and seasonal H3N2 virus resulted in increased replication and more severe pulmonary lesions in ferrets, whilst maintaining the capacity of airborne transmission [227]. However, this is yet to be recorded in humans. Future reassortment events are not unlikely since at least three of the four last pandemic influenza viruses occurred upon reassortment events between viruses of diverse origin.

Whereas the influenza virus HA has an important role in determining the virus host range and virulence, HA is also the main target of neutralizing antibodies. As a result, amino acid substitutions in the HA protein can result in escape from antibody-mediated neutralization [228]. This process is referred to as antigenic drift, and allows the virus to re-infect individuals that have acquired immunity to previously circulating strains through infection or vaccination. Therefore, once a new virus is established in the human population, antigenic variants are expected to emerge as a result of increasing population immunity. Consistent with this notion, it was recently shown that single amino acid substitutions near the RBS were sufficient to cause major antigenic changes during evolution of H3N2 and H5N1 [229], [230]. Thus, humans are not only susceptible to infection with influenza viruses from a variety of different sources, but these viruses continue to mutate and reassort in a constant battle to ensure efficient transmission and evasion of the host immune response.

## Conclusions

The ecology of influenza virus infections described in this review exemplifies the need for, and benefits of, a one-health approach to infectious disease. Indeed, the chance of a human influenza virus infection or pandemic is the direct result of a dynamic interplay between animal health, environmental factors and the immune system of the human host. Interestingly, despite the wealth of different influenza virus subtypes that circulate in birds only a limited number of influenza virus subtypes have been detected in humans. This clearly demonstrates that there are barriers to inter-species transmission. Some of these barriers may relate to the virus itself. For example, specific mutations in avian viruses appear to be necessary in order to ensure efficient human-to-human transmission. Alternatively, certain environmental factors may determine the rate of inter-species transmission such as close contact with infected animals or contact with contaminated water.

The human-animal interface remains in a constant state of flux and as our activities continue to change so too will the risk of influenza virus infection and inter-species transmission. Indeed, it is perhaps not surprising that the increase in poultry and swine populations observed worldwide since the industrial revolution have been associated with an increased frequency of zoonotic influenza virus infections in humans [231]. Currently of particular concern are activities such as cock fights, illegal trading of birds, live poultry markets and using poultry faeces to feed fish — all of which have been associated with the spread of HPAI H5N1 viruses [232]. Approaches which seek to curb the prevalence of these activities may thus have direct benefits for human health. However, any attempts to limit such activities must be accompanied by financial reimbursement for the relevant stakeholders and take into account local cultural practices and norms [233]. Moreover, the success of such preventative measures will depend upon the perceived risk of an outbreak or pandemic [233]. Thus, one-health solutions must be accompanied by campaigns to increase awareness about the potentially global consequences of influenza virus zoonosis. Ultimately, it is important to recognise that our understanding of the reservoirs and transmission of influenza virus continues to change as the virus itself evolves. This highlights the need for not only sound and up-to-date experimental studies, but also extensive surveillance and the continued emphasis on the fact that that human health is directly and inextricably linked to the health of animals and the surrounding environment.
